# Dissection of Mouse Hippocampus with Its Dorsal, Intermediate and Ventral Subdivisions Combined with Molecular Validation

**DOI:** 10.3390/brainsci12060799

**Published:** 2022-06-18

**Authors:** Aneta Jaszczyk, Adrian M. Stankiewicz, Grzegorz R. Juszczak

**Affiliations:** 1 Department of Animal Behavior and Welfare, Institute of Genetics and Animal Biotechnology, Polish Academy of Sciences, 05-552 Jastrzebiec, Poland; a.jaszczyk@igbzpan.pl; 2 Department of Molecular Biology, Institute of Genetics and Animal Biotechnology, Polish Academy of Sciences, 05-552 Jastrzebiec, Poland; a.stankiewicz@igbzpan.pl

**Keywords:** hippocampus, mice, dissection, dorsal, intermediate, ventral

## Abstract

Many research methods applied in molecular neuroscience require the collection of hippocampal samples, but a still poorly recognized problem is contamination with the choroid plexus during brain dissection. Because of a distinct pattern of gene expression, its inclusion in brain samples can obscure or even confound conclusions drawn from molecular studies. Therefore, we tested our dissection method designed for removal of tissue contamination using expression of the transthyretin gene (*Ttr*) as a marker of the choroid plexus. Additionally, we also validated dissection of the entire hippocampus into its dorsal, intermediate and ventral subdivisions using the expression of *Trhr* and *Lct* genes as molecular markers of anatomical subdivisions. The PCR analysis showed that *Ttr* is expressed at a residual level in hippocampal samples that display an mRNA level several hundred lower than the adjacent control tissue colocalized with the choroid plexus. This indicates that the applied method for dissecting the hippocampus from a fresh brain allows for replicable removal of the majority of choroid plexus from hippocampal samples. In turn, differences in expression of *Lct* and *Trhr* confirmed the proper dissection of dorsal, intermediate and ventral subdivisions from fresh brain tissue. Therefore, a special emphasis on the removal of tissue contamination and avoidance of tissue distortions makes our protocol especially suitable for molecular experiments performed either on the entire hippocampus or its subdivisions.

## 1. Introduction

The hippocampus is involved in various processes ranging from learning and memory to control of emotions and motivation [1]. It is also responsive to stress hormones [2] and is implicated in pathogenesis of various disorders such as depression [3], post-traumatic stress disorder [4], schizophrenia [5], epilepsy [6] and neurodegenerative diseases [7]. Therefore, it is not surprising that the hippocampus is one of the most frequently studied brain areas in rodents. Many research methods applied in molecular neuroscience require dissection of the hippocampus, but a still poorly recognized problem is tissue contamination during brain dissection. This issue has been raised in the past by several authors, especially in the context of inadvertent inclusion of the choroid plexus [8,9,10], which is located in the vicinity of hippocampus but is poorly visible in a mouse brain. Because of a distinct pattern of gene expression [9], its inclusion in brain samples can obscure or even confound conclusions drawn from molecular studies [10,11]. Our recent meta-analysis showed that the transcriptomic signature of the choroid plexus is common in microarray and RNA-seq experiments because it is present in about 25% of rodent studies, and the brain area most frequently affected is the mouse hippocampus [11]. Despite the importance of this issue, there is no validated method enabling dissection of the hippocampus from fresh mouse brain with precision sufficient for reliable removal of the choroid plexus. An alternative approach providing perfect precision is laser microdissection from frozen brain slices [8], but this method is very expensive and time-consuming, which severely limits its application. Therefore, we tested our dissection method designed for removal of tissue contamination. The dissection precision was assessed using expression of the transthyretin gene (*Ttr*) as a marker of the choroid plexus [8,9]. Additionally, we also validated dissection of the entire hippocampus into its dorsal, intermediate and ventral subdivisions. This is important because of the functional heterogeneity of different parts of the hippocampus [1] and because there is no validated dissection protocol for fresh brains obtained from adult mice, despite the availability of molecular markers differentiating the hippocampus across the dorsal-to-ventral axis [1,12].

## 2. Materials and Methods

### 2.1. Animals

The assessment of dissection precision was performed on tissues obtained from 5 male mice (Swiss-Webster) that were 3.5 months old and weighed 35.7 ± 1.3 g (mean ± SEM). Mice were obtained from the breeding colony located at the Institute of Genetics and Animal Biotechnology (Jastrzebiec). From each mouse, we dissected two hippocampi that constituted separate samples and a part of brain tissue constituting a positive control for expression of choroid plexus marker gene (*Ttr*). The final number of samples was 5 in case of *Ttr* positive control samples and 9 in case of hippocampi (one hippocampus was lost due to dissection failure). The sample quality assessment was performed on spare tissues collected during project 2017/27/B/NZ2/02796, which was performed with the permission of the Second Local Ethical Committee in Warsaw (permit number: WAW2/090/2018) and in accordance with the Polish Act of 15 January 2015 on the protection of animals used for scientific and educational purposes and the 3Rr principle.

### 2.2. Dissection Tools and Materials

**Figure 1:**
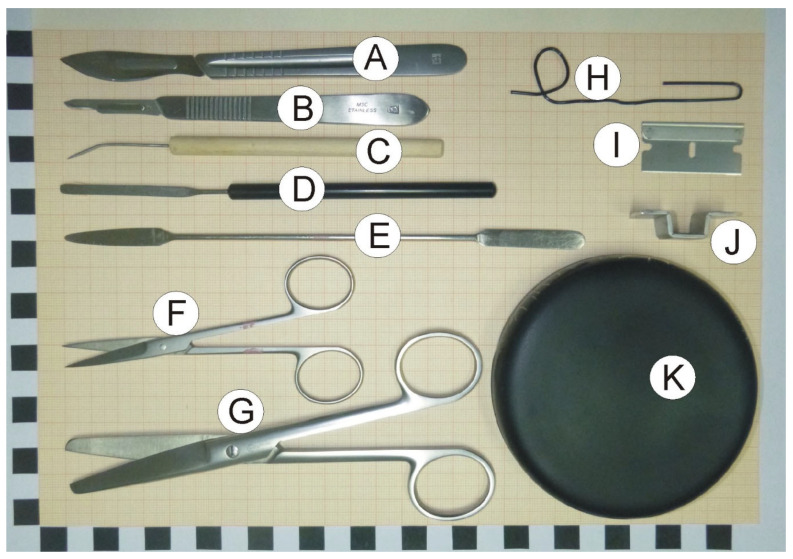
Dissection tools placed on millimeter paper. The black and white scale on the borders of the image is expressed in centimeters. For more information, please see Section 2.2.

- Scalpels with small (nb. 15) and large (nb. 24) blades (Figure 1A,B).
- Bent dissecting needle (Figure 1C).
- Stainless steel spatula with narrow blade (Figure 1D).
- Stainless steel spatula with flat round and tapered arrow ends (Figure 1E).
- Small surgical scissors (straight) with sharp tips (Figure 1F).
- Large surgical scissors (Figure 1G).
- A large paper clip that is used to prepare a loop restricting movement of dissected hippocampus at the time of rinsing with water (optional; Figure 1H).
- Single edge razor blade (optional; Figure 1I).
- Cutting form made from metal strip. The form helps to make precise vertical cuts (optional; Figure 1J).
- Convex cover of the Petri dish that serves as a dissection table. Convex surface is important because it enables water to flow out of the dissection surface. The cover can be painted black with mat waterproof paint to increase contrast between the background and the dissected tissue (Figure 1K).
- Wash bottle.
- Styrofoam box.
- Tabletop Illuminated Magnifier (3×).
- Filter paper.
- Millimeter paper.

### 2.3. Brain Dissection

**Figure 2:**
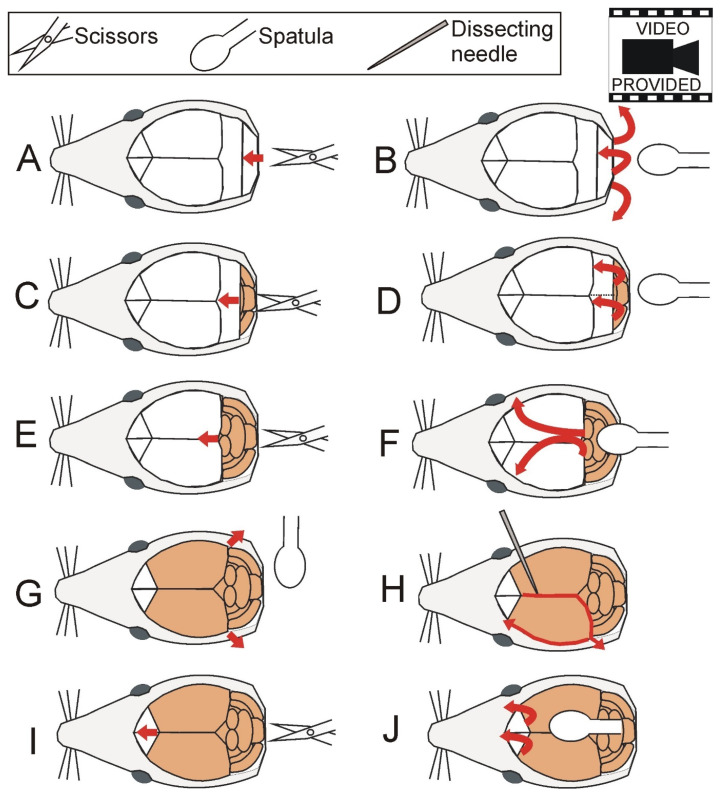
Removal of bones (**A**–**F** and **I**,**J**) and meninges (**H**). Additionally, bones on both sides of the brain (in the vicinity of occipital cortex) can be pulled apart (**G**) to facilitate disruption of the meninges and final brain removal. Bones are removed with small scissors and the round end of spatula while meninges are removed with a dissecting needle. Removal of meninges is shown only for one brain hemisphere for simplicity, but the procedure is performed on both hemispheres. The video is available in Supplementary Video S1 File.

Mice were euthanized by cervical dislocation. Head was separated from the rest of the body with large scissors while remnants of tissues (muscles and cervical vertebrae) were removed with small scissors. Skin covering the head was cut with small scissors along the midline starting from the occipital part to the interorbital constriction of the skull (approximately half of the total length of the skull). Muscles covering the skull were shoved aside with the round end of spatula. Next, bones were removed from the skull starting from the occipital part (Figure 2; Supplementary Video S1 File). The first cut was carried out by insertion of the tip of the small scissors into the foramen magnum (opening in the occipital bone of the skull), and bones were removed with the round end of spatula inserted gently under bones. The sequence of cutting and removing bones was repeated as illustrated in Figure 2 and Supplementary Video S1 File. At the time of cutting the bones covering hemispheres (Figure 2E,I), the lower tip of scissors should press the bones from the internal side of the skull so that the pressure is applied away from brain tissue. A crucial step is removal of meninges (Figure 2H) because they are very durable and easily damage brain. Frequently, the meninges are not visible after the removal of bones, although in some cases they are partly disrupted and therefore can be easily noticed at this stage of dissection (Supplementary Video S1 File). In both cases, meninges were removed as described below. The dissecting needle was slightly inserted into the interhemispheric fissure to pierce the meninges (or in front of already existing disruption) and moved in the posterior direction along the interhemispheric fissure and downward along the posterior edge of the hemisphere as indicated in Figure 2H. At the end of this movement, the needle was inserted deeply between the cortex and remnants of the skull and moved away from the brain to tear apart the meninges. Next, the needle was moved along the edge of the skull (Figure 2H), and the same procedure was repeated on the other side of the brain. After removal of all remaining bones (Figure 2I,J), the head was moved to vertical position and slightly tilted so the exposed brain was facing downwards. The nerves were cut with the tapered arrow end of the spatula starting from the occipital part of the brain. Finally, the spatula was inserted to separate olfactory bulb from olfactory nerves, and the brain was removed on a spatula (Supplementary Video S1 File).

### 2.4. Hippocampal Dissection

Dissection site constitutes Styrofoam box filled with crushed ice. The convex cover of the Petri dish that served as a dissection table was located on top of the ice. Convex surface is important because it enables water to flow out of the dissection surface. Depending on the need, the cover of the Petri dish can be turned around or tilted. Additionally, the cover of the Petri dish can be painted black (Figure 1K) to increase the contrast between dissected tissues and the background. The dissection was supported with the Tabletop Illuminated Magnifier (3×).

**Figure 3:**
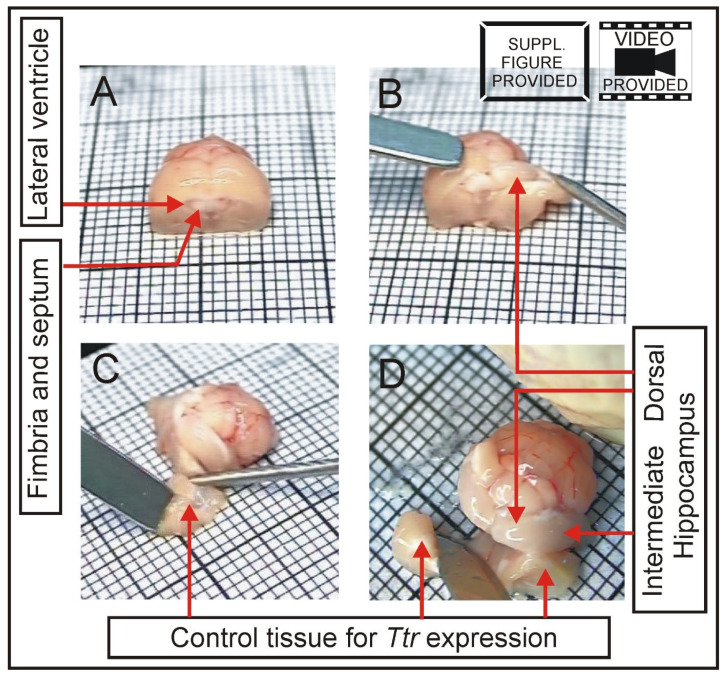
Removal of cortex from dorsal and ventral hippocampus and separation of control tissue colocalized with the choroid plexus to obtain positive control for expression of marker gene *Ttr*. (**A**) Posterior part of the brain after removal of frontal part. (**B**) Removal of cortex from dorsal hippocampus. (**C**) Cutting off the cortex. (**D**) Removal of tissues located in front of the hippocampus. The entire dissection protocol is presented in Supplementary Figure S1 and Supplementary Video S2 File.

**Figure 4:**
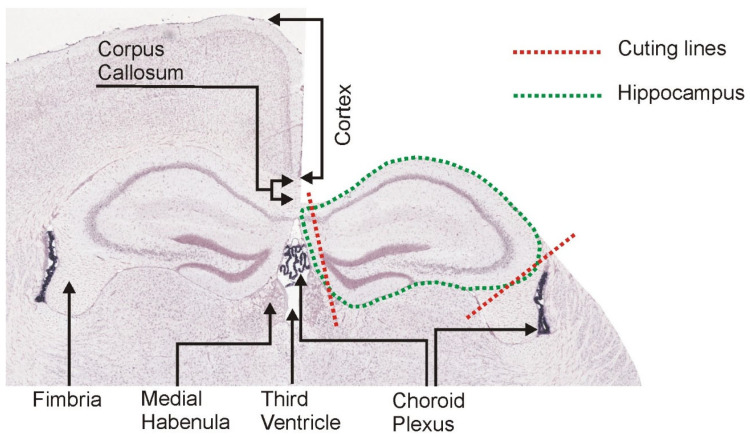
Coronal section of the mouse brain showing spatial relationship between dorsal hippocampus and choroid plexus. The in situ hybridization (ISH) image was derived from the Allen Mouse Brain Atlas (https://mouse.brain-map.org/, accessed on 4 January 2022) [13]. Part of the cortex (right side of the image) has been masked to visualize brain during the dissection process (Figure 3, Supplementary Figure S1 and Supplementary Video S2 File).

First, a square of filter paper was placed on the Petri dish lid to prevent sliding of the brain during dissection. The filter paper was soaked with ice-cold sterile water. The dorsal and ventral parts of the brain were rinsed thoroughly with ice-cold sterile water (Supplementary Figure S1A,B). Next, the olfactory bulb was removed with tapered arrow end of spatula (Supplementary Figure S1C). The brain was placed on a square of millimeter paper to make a vertical cut with single edge razor blade to separate the anterior part containing frontal cortex and the posterior part containing hippocampus, thalamus and hypothalamus. The cutting line was 3–4 mm from the frontal pole (Supplementary Figure S1D). The anterior part containing frontal cortex can be used for collection of other brain areas or discarded while the posterior part (Figure 3) is used for dissection of hippocampi. The cortex overlying dorsal hippocampi was removed with dissecting needle and spatula (with a narrow blade) starting from the interhemispheric fissure (Figure 3, Supplementary Figure S1H–J). The removal of cortex was preceded by partial disruption of the corpus callosum (Supplementary Figure S1D). The spatula was placed gently on the cortex from one side of the fissure to hold the brain in place at the time when the cortex on the other side was shoved aside with a dissecting needle (Figure 3B, Supplementary Figure S1I). Next, the spatula was placed on partly removed cortex and the needle was used to shove aside contralateral cortex (Supplementary Figure S1J). Released parts of cortex were cut off (Figure 3C, Supplementary Figure S1K,L) and collected into vials to freeze in liquid nitrogen. In our experiment, these parts of cortex were included in the control tissue (Figure 3C) for measurement of *Ttr* expression. Next, a cut was performed with the tip of the scalpel along the posterior and anterior edges of the hippocampi to separate them from white matter (Supplementary Figure S1N,O). Finally, the white matter located in front of the hippocampi was completely removed together with the remaining brain tissue (Figure 3D, Supplementary Figure S1P) and saved for further analysis. In our experiment, this part of brain was included in the control tissue together with cortex removed earlier (Figure 3C,D) for measurement of *Ttr* expression. Next, dorsal parts of hippocampi were separated from tissues located in the third ventricle or its vicinity with angled cuts (Figure 4) that were perpendicular to the longitudinal axis of the hippocampus (Supplementary Figure S1R). At this stage, the dissection exposed dorsal and intermediate parts of both hippocampi (Figure 3D) while the ventral part was still covered with the cortex. To expose the ventral hippocampus, it is necessary to separate the hemispheres (Supplementary Figure S1S) and to roll over the brain to the medial surface created by the cut (Supplementary Figure S1T,U). The border between the ventral hippocampus and the cortex may not be well visible. Therefore, this part of brain was thoroughly rinsed with the stream of ice-cold sterile water to separate the cortex from the hippocampus (Supplementary Figure S1V). Partly detached cortex can be additionally pushed away with a dissecting needle and cut off with scalpel. Final incision was performed along the exposed ventral hippocampus (Supplementary Figure S1W) and between the anterior edge of hippocampus and tissues located beneath (Supplementary Figure S1X). The hippocampus was rolled over with dissecting needle (Supplementary Figure S1Y,Z) and detached from other brain tissues with the stream of ice-cold water (Supplementary Figure S1AA). Partly detached cortex was additionally pushed away with a dissecting needle in case not all connections were disrupted during earlier steps. The hippocampus was picked up with spatula (Supplementary Figure S1AB) and placed on clean paper filter soaked with the ice-cold sterile water for final removal of tissue contamination (Supplementary Figure S1AC–AE). At this stage, all remnants of white matter and cortex were removed with the scalpel. The stream of water was used to remove and visualize remnants of tissues attached to the dorsal and ventral surfaces of the hippocampus (Supplementary Figure S1AC). Wire loop can be used to keep the hippocampus in place during thorough rinsing (Supplementary Figure S1AE). The procedure was performed until there were no strings or pieces of tissue visible at the time of rinsing the hippocampus. Finally, the hippocampus was separated into three equal parts (Supplementary Figure S1AF) corresponding to the dorsal, intermediate and ventral subdivisions (Supplementary Figure S1AF) with the help of the millimeter paper and the scalpel. The dorsal part can be easily recognized by its shape (Supplementary Figure S1AF) resulting from the perpendicular cut performed in earlier step of the procedure (Supplementary Figure S1R). Obtained samples were frozen in liquid nitrogen and stored at −80 °C.

### 2.5. Real-Time PCR (PCR)

Total RNA was extracted from the individual samples using GeneMATRIX universal RNA purification kit (Eurx). The quantity and quality of all RNA samples were assessed by spectrophotometry (ND-1000, Nanodrop). To verify the precision of dissection, we analyzed expression of marker genes *Trhr*, *Lct* and *Ttr* together with reference gene *Hmbs*. The expression was analyzed with SYBR Green-based qPCR performed in 96-well plates on the Roche LightCycler^®^ 96 thermocycler. The primers used in qPCR were designed with the Primer-BLAST tool. The designed primers were located on two different exons and contained all mRNA transcripts of each specific gene. The annealing temperature for individual primers was determined by performing PCR with a set temperature gradient (55°–65°) during 3-step amplification. Primer specifications are presented in Table 1. For retrotranscription into cDNA, 500 ng of total RNA from each sample was used (First Strand cDNA Synthesis Kit, Roche). qPCR was performed on the FastStart Essential DNA Green Master kit (Roche) according to the protocol provided by the manufacturer. All genes were run in three replicates, and each repetition was performed on a separate plate. Each plate contained two negative controls (without cDNA) and a series of 5-fold dilutions of the total cDNA sample to determine PCR efficiency. The reaction volume was 20 µL (*Lct*, *Ttr* and *Hmbs* genes) or 40 µL (*Trhr* gene). The relative expression of marker genes was calculated using Pfaffl method [14].

### 2.6. Statistics

Raw and square root transformed data [15] were first tested for variance homogeneity with C Cochran, Hartley, Bartlett test. The analysis showed that the data did not meet the requirement of variance homogeneity even after square root transformation. Therefore, we used nonparametric Mann–Whitney U test to compare expression in adjacent parts of the hippocampus (dorsal vs. intermediate and intermediate vs. ventral) in case of molecular markers of hippocampal subdivisions and between hippocampus and control tissue in case of molecular marker of choroid plexus. Data analysis was performed with Statistica software, release 7.1. Values are presented as mean ± SEM (column bar graphs) and scatter plots.

## 3. Results

### 3.1. Expression of Choroid Marker Gene Ttr

**Figure 5:**
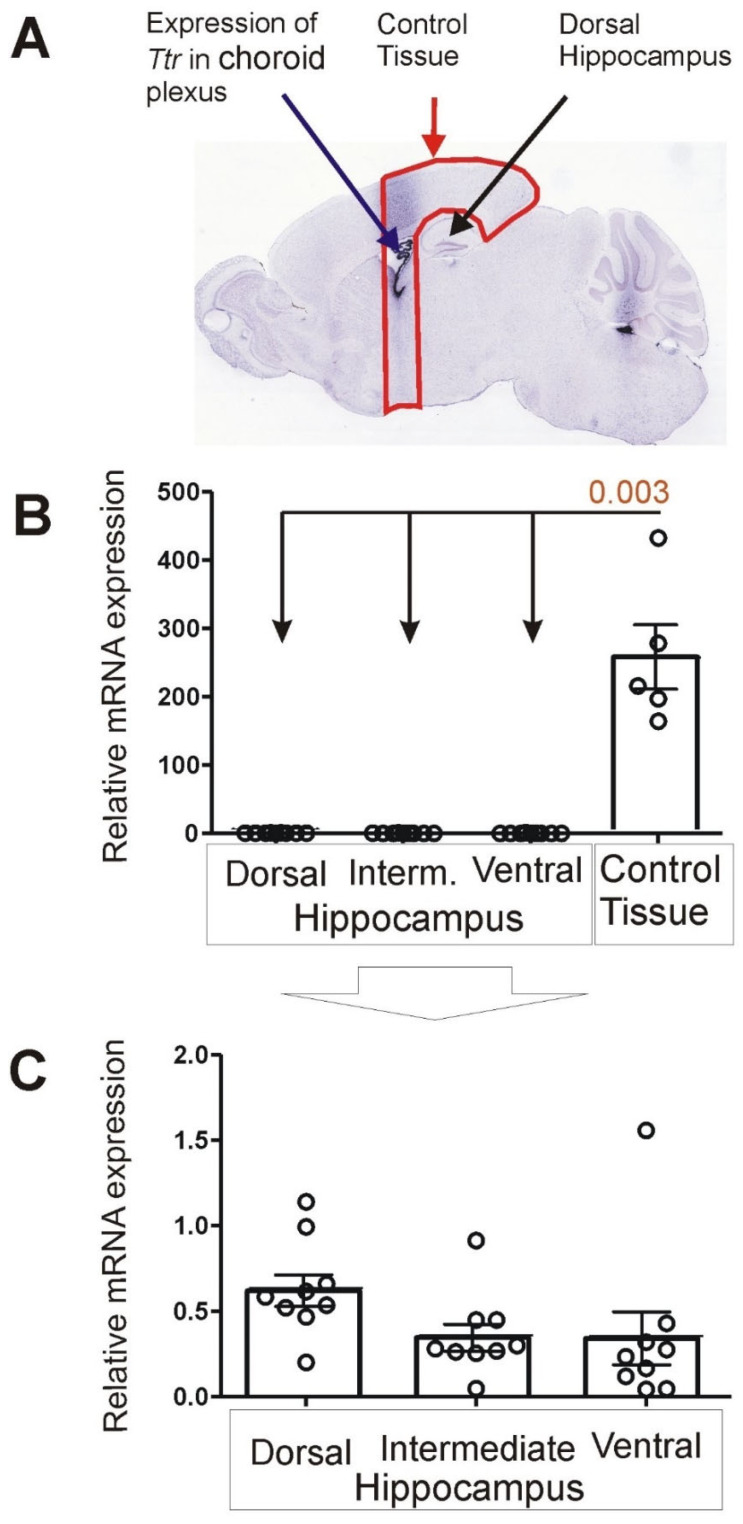
Brain expression of *Ttr*. (**A**) Pattern of expression in choroid plexus and dorsal hippocampus (ISH image) derived from Allen Mouse Brain Atlas (https://mouse.brain-map.org/, accessed on 23 June 2021) [13]. (**B**) Results of PCR analysis performed on control tissue and dissected samples of dorsal, intermediate and ventral hippocampus. (**C**) Details of *Ttr* expression in hippocampal subdivisions presented with altered scale at the Y axis. Data are presented as mean ± SEM (column bar graphs) overlaid on scatter plots.

Real-time PCR analysis revealed very low expression of the Ttr gene in all parts of the hippocampus (dorsal, intermediate and ventral; *n* = 9) in contrast to the control tissue (*n* = 5) composed of brain tissue located in front of the hippocampus and part of the cortex overlying the hippocampus (Figure 5). Mean expression was about 400 to 700 times higher in the control tissue compared with subdivisions of dissected hippocampi. The differences were significant for all parts of the hippocampus compared with the control (*p* = 0.003).

### 3.2. Expression of Marker Genes Differentiating between Dorsal and Ventral Hippocampus

**Figure 6:**
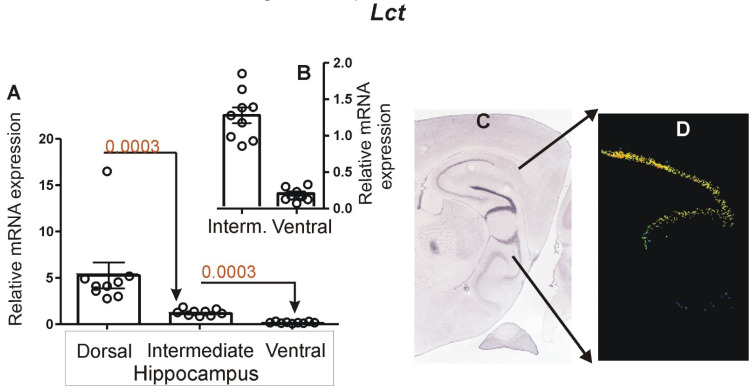
Hippocampal expression of *Lct*. (**A**) Results of PCR analysis performed on dissected samples of dorsal, intermediate and ventral hippocampus. (**B**) Details of *Lct* expression in intermediate and ventral parts presented with altered scale at the Y axis. (**C**) Pattern of expression in brain slice (ISH image) containing all hippocampal subdivisions derived from Allen Mouse Brain Atlas (https://mouse.brain-map.org/, accessed on 24 June 2021) [13]. (**D**) Expression detection mask retrieved from Allen Mouse Brain Atlas showing dorsal hippocampus with highest *Lct* expression. Data are presented as mean ± SEM (column bar graphs) overlaid on scatter plots.

Real-time PCR analysis showed that expression of *Lct* was highest in the dorsal hippocampus (*n* = 9) and lowest in the ventral hippocampus (*n* = 9), while the intermediate part (*n* = 9) displayed an intermediate level of expression (Figure 6). The differences between adjacent parts of hippocampus (dorsal vs. intermediate and intermediate vs. ventral) were significant with *p* = 0.0003.

**Figure 7:**
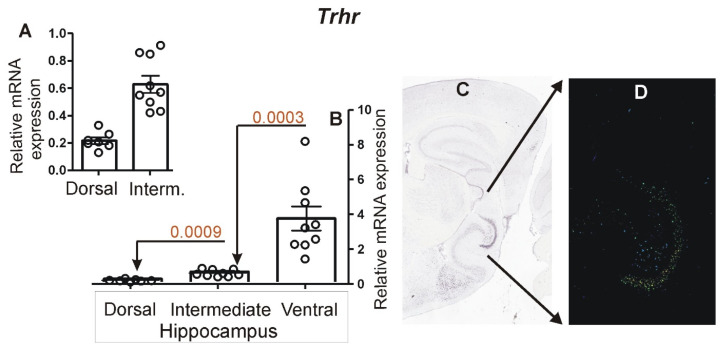
Hippocampal expression of *Trhr*. (**A**) Details of *Trhr* expression (PCR) in dorsal and intermediate parts presented with an altered scale at the Y axis. (**B**) Results of PCR analysis performed on dissected samples of dorsal, intermediate and ventral hippocampus. (**C**) Pattern of expression in a brain slice (ISH image) containing all hippocampal subdivisions derived from Allen Mouse Brain Atlas (https://mouse.brain-map.org/, accessed on 24 June 2021) [13]. (**D**) Expression detection mask retrieved from Allen Mouse Brain Atlas showing mediodorsal hippocampus with highest *Trhr* expression. Data are presented as mean ± SEM (column bar graphs) overlaid on scatter plots.

The *Trhr* gene displayed an opposite pattern of expression characterized by the highest level of expression in the ventral hippocampus (*n* = 9) and lowest in the dorsal hippocampus (*n* = 7) as indicated by the real-time PCR analysis (Figure 7). In the case of the two samples from the dorsal hippocampus, we have obtained negative results for all repeats of the PCR analysis (triplicate). Because of an uncertainty about the reasons for the negative results (technical error vs. lack of expression), we omitted these two samples from the statistical analysis, reducing the total number of samples to seven. The intermediate part (*n* = 9) displayed an intermediate level of expression of *Trhr*. The differences were significant, with *p* = 0.0009 (dorsal vs. intermediate part) and *p* = 0.0003 (intermediate vs. ventral part).

## 4. Discussion

Our dissection method is different than most of the other gross dissection protocols that require initial separation of hemispheres before exposition of the hippocampus [16,17,18,19,20,21]. Most of these protocols also require complete removal or displacement of the brainstem and diencephalon (thalamus and hypothalamus) to access the hippocampus from the inside of the mouse or rat brain (Table 2). As a result, dissection of the hippocampus is preceded by disintegration of the brain, which makes such dissection methods less intuitive and obscures the spatial relationships between the hippocampus and the rest of brain. In contrast, our method relies on removal of the cortex starting from the dorsal part of the hippocampus before the separation of the hemispheres and therefore makes it easy to observe spatial relationships in brain anatomy (Figure 3, Supplementary Figure S1 and Supplementary Video S2 File). In general principle, our protocol resembles the approach used previously by Spijker [22], although the details of these protocols are different in many respects. The unique feature of our protocol is the angled cuts used to separate the right and left hippocampus (Figure 4) without collection of the choroid plexus located between them in the third ventricle (Figure 4). Other protocols apply a single cut along the midline of the brain [16,17,18,19,20,21] or disrupt this part with forceps [22] without removal of tissues located between hippocampi. The second distinctive feature of our protocol is the usage of a stream of water to minimize damage or distortion of hippocampi at the time of the tissue separation and to remove potential tissue contaminations. Finally, we put a special emphasis on the removal of all remnants of white matter along the edges of hippocampi as a part of an effort to remove tissue contamination that can be contributed to, for example, by fimbria with the adjacent choroid plexus (Figure 4). To verify the effectiveness of our protocol, we used the transcriptomic marker of the choroid plexus [8,9]. The PCR analysis showed that the transthyretin gene (*Ttr*) is expressed at a residual level in hippocampal samples that display an mRNA level several hundred lower than adjacent control tissue colocalized with the choroid plexus (Figure 5) consistently with our previous assessment based on analysis of brain slices [9]. This indicates that the applied method for dissecting hippocampus from fresh brain allows for replicable removal of majority of choroid plexus from hippocampal samples. Therefore, the presented dissection method is especially suitable for molecular studies performed on homogenized tissues that are sensitive to contamination [9,10,11]. Such tissue contamination can be responsible not only for false positive findings that are present in many published datasets but may also obscure genuine changes in expression of some genes shared between tissues [11]. Importantly, no other gross dissection protocol for mice or rats (Table 2) has tested the presence of contaminations in collected samples, and most of these papers [16,17,18,19,20,22] do not even mention the fact that the choroid plexus is in dissected brain tissue. This issue is also neglected in protocols describing free-hand dissection of rat and mouse hippocampi from brain slices [23,24]. The only available alternative that was proved to be effective in the removal of the choroid plexus is laser microdissection [8].

It should be noted that some research methods may not be affected by contamination. An example is electrophysiology performed on slices, which is associated with the precise localization of electrodes under microscopic control together with the characterization of the electrical properties of recorded cells. In fact, precise removal of the choroid plexus before electrophysiological experiments is a waste of time. Instead, crucial factors for such studies are the speed of dissection determining the viability of cells and the preservation of specific neural circuits. Therefore, the selection of dissection approach depends on the aim of the experiment, and some available protocols were designed specifically for electrophysiology performed in mouse and rat hippocampal slices with different planes of cutting [18,20,25,26]. Some of these protocols use larger parts of the mouse [18,25] or rat [26] brain to cut slices containing both the hippocampus and surrounding tissues.

We also showed that our dissection method is convenient for separating dorsal, intermediate and ventral hippocampus. This is important because of functional and transcriptomic differences between these subdivisions [1,12,27,28]. Some special aspects of our protocol make it especially suitable for this purpose. Gross dissections applied previously relied on cutting the hippocampus into three equal parts corresponding to dorsal, intermediate and ventral subdivisions [27] or just into two parts without precise specification of borders between them [28]. Importantly, a precise separation of these subdivisions depends on dissection of the entire hippocampus and preservation of its size. This is crucial because fresh brain tissue is soft and malleable and therefore can be easily damaged or distorted during dissection, leading to serious errors at a time when different parts of hippocampus are attributed to subdivisions observed in an intact brain. Therefore, we are not using forceps or tweezers to prevent changes in the shape and length of the hippocampus. Instead, we rely on using a stream of water to separate hippocampus from adjacent brain areas. Second, the dorsal and ventral part can be easily mistaken when the hippocampus is removed from the rest of the brain and manipulated for removal of remnants of other tissues. This problem is avoided in our protocol because we apply cuts that are perpendicular to the longitudinal axis of the hippocampus (Supplementary Figure S1R) to separate its dorsal part from tissues located in the third ventricle (Figure 4). Additionally, these cuts differentiate between the dorsal and ventral ends of dissected hippocampi (Supplementary Figure S1AF) and therefore facilitate dissection of hippocampal subdivisions. The sufficient precision of this approach has been confirmed by the expression of *Lct* and *Trhr* genes (Figure 6 and Figure 7) that are known as molecular markers of the dorsal and ventral hippocampus identified previously in brain slices [1,12]. It means that our gross dissection of fresh brain recapitulated observations of differential gene expression detected in brain slices [1,12] 

It should be noted that our protocol can be easily combined with dissection methods relying on cutting slices from fresh brain with tissue choppers or special forms to obtain various brain regions [23,29]. Alternatively, some gross dissection protocols for collection of multiple brain areas can also be included [16,21,22]. Such a combination of methods will allow for dissection of additional brain areas located between the frontal pole and the hippocampus. A special emphasis on the removal of tissue contamination and avoidance of tissue distortions makes our protocol especially suitable for molecular experiments performed either on the entire hippocampus or its subdivisions. Therefore, this provides an alternative to laser microdissection from frozen slices [8], which requires expensive equipment and is time consuming.

## Figures and Tables

**Table 1:** Primers used for PCR validation of dissection precision.

Gene Name	Forward or Reverse Primer	Primer Sequence	Annealing Temperature	Efficiency
*Trhr*	F	GAGCCTCTGCTAAGTGATCTTCC	58°	97%
	R	ACGGGGACTCTAAAACATCTTTCC		
*Lct*	F	CGTCAGCCAAGGTCTACGC	60°	93.7%
	R	GTCTGTGCTTCTGCCGTGC		
*Ttr*	F	TCGCGGATGTGGTTTTCACAG	60°	106.2%
	R	CTCTCAATTCTGGGGGTTGCT		
*Hmbs*	F	TCCTGGCTTTACTATTGGAG	60°	95.2%
	R	TGAATTCCAGGTGGGGGAAC		

**Table 2:** Summary of available protocols for gross dissection of hippocampus or its parts in rodents.

Author	Entire Hippoc.	Subparts	Species	General Dissection Strategy	Video	Application
[16]	Yes	No	Rats	Separation of hemispheres and removal of the brainstem/diencephalon to expose lateral ventricle and medial side of the hippocampus	Yes	General
[18]	Yes	No	Rats	Separation of hemispheres and displacement of the brainstem/diencephalon preceding the exposition of lateral ventricle and medial side of the hippocampus	Yes	Electro- physiol
[17]	No	Dentate gyrus	Mice	Separation of hemispheres and removal of the brainstem/diencephalon to expose the lateral ventricle and medial side of the hippocampus	Yes	General
[20]	Yes	No	Mice	Separation of hemispheres and removal of the brainstem/diencephalon to expose the lateral ventricle and medial side of the hippocampus	Yes	Electro- physiol
[21]	Yes	No	Mice	Separation of hemispheres and eversion of the lateral ventricle	Yes	General
[19]	Yes	CA1, CA3 and Dentate gyrus	Mice	Separation of hemispheres and removal of occipital cortex starting from lateral side of the brain	No	Genera
[22]	Yes	No	Mice/ Rats	Removal of occipital cortex starting from the dorsal part of the brain	No	General

## Data Availability

All data are provided in the form of scatter plots in the manuscript.

## Supplementary Materials

The following supporting information can be downloaded at: https://zenodo.org/record/6515868#.YnJCb4fP2DJ (Doi:10.5281/zenodo.6515868); Video S1—procedure for removal of mouse brain; Video S2—procedure for collecting hippocampus; Figure S1—procedure for collecting hippocampus.
